# The Prognostic Value of DCE-MRI Findings before Salvage Radiotherapy after Radical Prostatectomy

**DOI:** 10.3390/cancers15041246

**Published:** 2023-02-15

**Authors:** Alessia Farneti, Marta Bottero, Adriana Faiella, Diana Giannarelli, Luca Bertini, Valeria Landoni, Patrizia Vici, Pasqualina D’Urso, Giuseppe Sanguineti

**Affiliations:** 1 Radiation Oncology, IRCCS Regina Elena National Cancer Institute, 00144 Rome, Italy; 2 Biostatistics, IRCCS Regina Elena National Cancer Institute, 00144 Rome, Italy; 3 Radiology, IRCCS Regina Elena National Cancer Institute, 00144 Rome, Italy; 4 Physics, IRCCS Regina Elena National Cancer Institute, 00144 Rome, Italy; 5 Phase IV Studies, IRCCS Regina Elena National Cancer Institute, 00144 Rome, Italy

**Keywords:** prostate cancer, salvage radiotherapy, dynamic contrast enhancement magnetic resonance imaging, local failure, biochemical failure

## Abstract

**Simple Summary:**

The role of restaging before salvage radiotherapy (sRT) for prostate cancer is still controversial. The aim of the present retrospective study was to investigate the predictive value of dynamic contrast-enhanced-magnetic resonance imaging (DCE-MRI) findings, including the volume and the subsite of the presumed local failure. We found that DCE-MRI at restaging for biochemical failure after radical prostatectomy seems to provide both predictive and therapeutic information. Patients with small lesions at the vesico-urethral anastomosis have an excellent prognosis after sRT.

**Abstract:**

Background: To investigate the predictive role of dynamic contrast-enhanced-magnetic resonance imaging (DCE-MRI) findings before salvage radiotherapy after radical prostatectomy (RP). Methods: This retrospective study selected patients with biochemical failure (BF) after RP restaged with DCE-MRI. Patients underwent sRT in 30 fractions delivering 66–69 Gy and 73.5 Gy to the prostatic fossa and to the local failure as per DCE-MRI, respectively. Pelvic nodes were treated to 54 Gy in selected patients. The endpoint was BF after sRT. Results: In total, 236 patients were analyzed and 146 (61.9%) had presumed local failure at DCE-MRI: 54.8%, 23.8% and 21.4% were found at the vesico-urethral anastomosis (VUA), the bladder neck and the retro-vesical space, respectively. The presence of a local failure at DCE-MRI halved the risk of BF; VUA-only location and lesion volume were independently correlated with survival without evidence of biochemical failure (bNED) at multivariable analysis. For patients with VUA-only disease up to 0.4 cc, the 4-year-bNED was 94.6% (95%CI: 80.2–98.6%) as opposed to 80.9% (95%CI: 71.6–87.4%) and 73.7% (95%CI: 63.1–81.8%) for other lesions and no macrodisease, respectively. Conclusions: DCE-MRI at restaging for BF after RP provides predictive and therapeutic information. Patients with small lesions at the VUA have an excellent prognosis after sRT.

## 1. Introduction

Salvage radiotherapy (sRT) provides the possibility of a cure for patients with a biochemical failure after radical prostatectomy (RP) [1,2]. The premise of successful sRT is that all disease is confined within the treated volumes. Therefore, restaging before sRT has to rule out distant metastases [3,4], and to this end, prostate-specific membrane antigen positron emission tomography/computed tomography (PSMA PET/CT) is the imaging modality with the highest sensitivity in the setting of early sRT [5]. 

Whether the detection of disease in the prostatic fossa provides any benefit is controversial. Proponents of withholding local restaging claim that there is no evidence of a clinical gain for sRT dose escalation to the prostatic fossa [6,7]. However, the detection of local disease allows selective boosting/dose escalation [8] and provides prognostic information as well [9,10,11]. Indeed, according to a recent retrospective study from the Mayo Clinic, patients with presumed local disease at dynamic contrast enhanced-magnetic resonance imaging (DCE-MRI) have a more favorable outcome than patients with negative findings [9]. Unfortunately, PET CT even with PSMA has a significantly poorer local detection rate than DCE-MRI [12,13]. Therefore, if local restaging is performed, DCE-MRI has to be added to the patient’s work-up. 

Since, at our institution, DCE-MRI has been routinely offered to all patients before sRT, the purpose of the present study is to investigate the predictive role of DCE-MRI findings, including volume and the subsite of the presumed local failure. 

## 2. Materials and Methods

### 2.1. Patients

All patients referred for sRT to our institution with a biochemical failure [14] after RP have been consistently offered restaging with both PET/CT and multiparametric MRI since 2014. The present study is an Institutional Review Board-approved (RS1660/22), single-institution, retrospective study focusing on patients without regional/distant disease at PET/CT to investigate the predictive role of (local) DCE-MRI findings on outcome after sRT. Exclusion criteria were history of androgen deprivation (AD) at any point before sRT and pathologically positive nodes at initial surgery. 

### 2.2. Restaging Work-Up

All included patients underwent both multiparametric MR of the pelvis and PET/CT with choline and/or PSMA. Details of both DCE-MRI and PET/CT techniques have been reported previously [12,15,16]. Workup performed at an outside institution was considered only if the whole exam was available for review and, for DCE-MRI only, it satisfied selected technical criteria, including the use of a 3 tesla (3T) magnet, the presence of DCE sequence and the lack of endorectal coil. A proportion of patients underwent multiple tracer PET/CTs in a prospective study [12].

DCE-MRI scans were read by a single observer (LB) and defined as positive in case of an early/fast enhancing discrete lesion possibly accompanied by a hyperintense soft tissue on T2 weighted images as previously reported [15]. Patients with evidence of macroscopic disease outside the prostatic bed (pelvic nodes or distant) were excluded. The location of the local failure was identified as per Connolly et al. [17]. Each discrete local lesion was contoured and the volume computed as reported elsewhere [18].

### 2.3. Treatment

All patients underwent 3T DCE-MRI without an endorectal coil and the lesion(s) were transferred to the planning CT after co-registration as previously reported [18]. The sRT consisted in 73.5 Gy to the presumed local lesion and 66–69 Gy to the prostatic bed in 30 fractions (fxs) with a simultaneous integrated boost technique. Pelvic nodes (PN) were covered to 54 Gy/30 fxs in selected patients [15].

### 2.4. Endpoints

The endpoint of the study was the development of a biochemical failure defined as a 0.2 ng/mL prostate-specific antigen (PSA) rise above the nadir after sRT or the initiation of salvage AD therapy [10]. Patients without a biochemical failure were censored at the date of the last PSA. Time to biochemical failure was calculated from the date of sRT end. 

### 2.5. Statistical Analysis

Actuarial survival curves were computed with the Kaplan–Meier method. Various covariates (age, pre-RP PSA, pathological (p) tumor (T) and p nodal (N) stages at RP, margins status at RP, International Society of Urological Pathology (ISUP) grade group, time from RP to sRT, PSA doubling time (PSADT), PSA detectability after RP, PSA at sRT; the location, number and volume of the detected recurrence(s), AD use, PN coverage, European Association of Urology (EAU) risk category) were investigated at univariable analysis (UVA) on the time to biochemical failure (bNED-survival). All covariates with a *p* value < 0.2 at UVA were subjected to a Cox proportional hazards regression analysis.

Groups were compared with the chi-squared test, the Mann–Whitney rank test, or the log-rank test when appropriate. For proportions, confidence intervals (CI) were computed with the Wilson score method without continuity correction. 

All statistical tests were performed using GraphPad (version 8.0.1, GraphPad Software Inc., San Diego, CA, USA) and SPSS (version 25, IBM, Armonk, NY, USA).

## 3. Results

From January 2014 to June 2020, 236 patients underwent sRT after being restaged by both PET/CT and DCE-MRI for biochemical failure after RP. Selected patients, tumor and treatment characteristics are reported in Table 1. 

All but 11 patients underwent choline PET/CT, while PSMA PET/CT was performed on 82 (34.7%) patients; overall, about 1/3 of the patients underwent more than one PET/CT tracer at restaging. All patients were without evidence of disease in the regional nodes as well as distantly before sRT.

**Figure 1:**
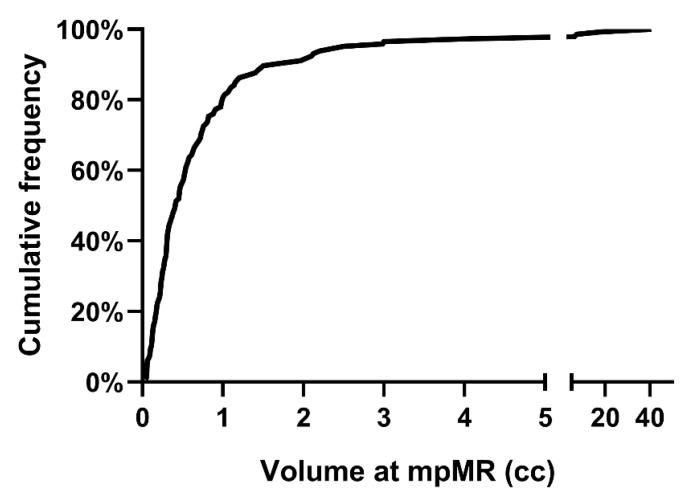
Cumulative frequency of the volume of local disease at mpMRI for patients with positive DCE-MRI. Abbreviations: mpMRI: multiparametric magnetic resonance imaging; DCE-MRI: dynamic-contrast-enhanced magnetic resonance imaging.

Ninety patients (38.1%) were not found to harbor macroscopically evident local disease at DCE-MRI. Out of 146 patients with detected local disease, 125 (85.6%), 20 (13.7%), and 1 (0.7%) patient had one, two, or three discrete lesions at DCE-MRI, respectively. Therefore, 168 local lesions have been detected overall, 92 (54.8%), 40 (23.8%) and 36 (21.4%) at the vesico-urethral anastomosis (VUA), the bladder neck (BN) and the retro-vesical (RV) space, respectively. The cumulative frequency of the volume of disease at DCE-MRI for each patient with a positive DCE-MRI is reported in Figure 1.

There was no correlation between the subsite location of the failure and its volume (*p* = 0.959). Median PSA was significantly smaller in patients with positive compared with negative DCE-MRI findings (0.46 ng/mL vs. 0.60 ng/mL, *p* = 0.015). Among the subsites, serum PSA was not different between VUA-only and RV/BN ± VUA lesions (*p* = 0.305).

The involvement of the RV region was significantly more frequent in patients with multiple rather than single lesions (66.7%, 95%CI: 45.4–82.8% vs. 17.6%, 95%CI: 11.9–25.2%, *p* < 0.001), while this was not the case for both VUA (61.9%, 95%CI: 40.9–79.2% vs. 57.6%, 95%CI: 48.8–65.9%, *p* = 0.137) and BN (38.1%, 95%CI: 20.8–59.1% vs. 24.8%, 95%CI: 18.1–33.0%, *p* = 0.203) subsites. Overall, 79 patients (33.5%) had isolated involvement at the anastomosis; the remaining 67 patients (28.4%) had disease at the BN/RV spaces ± the VUA. 

All patients received intensity-modulated sRT in 30 fractions. For patients with local disease, the dose to the detected local recurrence was 73.5 Gy in all patients but 9 (6.3%) who received 72 Gy (N = 5) and 69 Gy (N = 4), respectively. The prostatic fossa received a median dose of 69 Gy, although few patients received a lower dose (Table 1). Pelvic nodes were covered in 150 patients (63.6%). A total of 11 (4.7%) and 14 (5.9%) patients received short-term (6 months (mths)) or long-term (2 years) androgen deprivation as well. 

### bNED Rates & Predictors of Response

**Figure 2:**
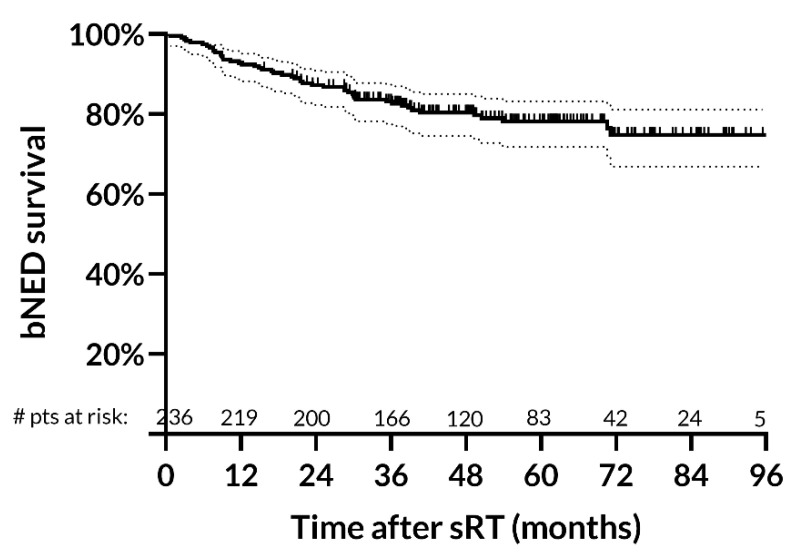
Biochemical survival rates after salvage radiotherapy. Four-year biochemical control rate is 80.4% (95% CI: 74.5–85.1%). Abbreviations: bNED: biochemical no evidence of disease; sRT: salvage radiotherapy.

After a median follow-up of 50.9 months (IQR: 36.9–67.8 months), we observed 49 biochemical failures at a median time of 20.1 months (IQR: 8.8–30.2 months) from the treatment end. Four-year biochemical control rate was 80.4% (95%CI: 74.5–85.1%) (Figure 2). 

Results of univariable analysis on bNED survival are reported in Supplementary Table S1. The following variables were entered at multivariable analysis (MVA): DCE-MRI status (positive vs. negative); disease volume (continuum); EAU risk (low vs. high); seminal vesicle invasion (no vs. yes); serum PSA before RP (continuum); detectable PSA after RP (continuum); androgen deprivation (yes vs. no). PSADT was not entered at MVA because it is already included within the EAU risk stratification. Disease subsite (none vs. VUA-only vs. BN/RV ± VUA) was not included in the initial analysis at MVA. As shown in Table 2, the presence of a local failure at DCE-MRI halved the risk of biochemical failure after sRT. 

When this covariate was replaced by the subsite of failure at DCE-MRI, VUA-only location was independently correlated to bNED survival with a risk of about 1/3 of the one having no detectable local disease at DCE-MRI. However, the outcome of patients with cranial involvement at the bladder’s neck/retro-vesical space was not statistically different from the one of patients without detectable lesions.

Moreover, at both MVA analyses, the volume of the detected lesion was highly significant with a decrease in the probability of survival without biochemical failure of around 6–7% for every cc of disease detected at DCE-MRI. EAU risk stratification and SVI invasion had an inconsistent/borderline correlation to bNED survival (Table 2), while serum PSA before RP, detectable PSA after RP and androgen deprivation were not significantly correlated with bNED survival.

**Figure 3:**
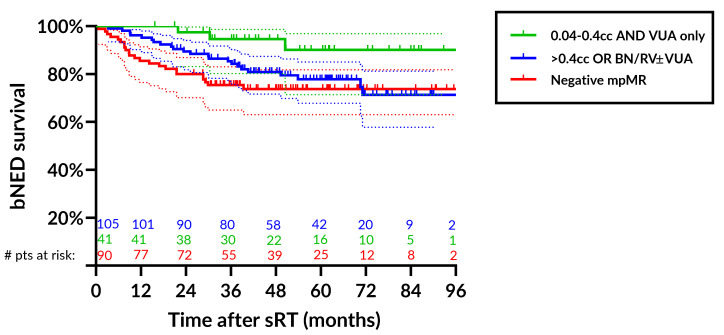
Biochemical control rates after salvage radiotherapy stratified by the location of the lesion (none, VUA-only, BN/RV ± VUA) and by the volume of the lesion at DCE-MRI using as a cutoff a median value of 0.4 cc. For patients with VUA-only disease up to 0.4 cc, the bNED rate at 4 yrs was 94.6% (95%CI: 80.2–98.6%) (green line), for patients with >0.4 cc or other detectable lesions (BN/RV ± VUA), the 4 yr bNED rate was 80.9% (95%CI: 71.6–87.4%) (blue line) and 73.7% (95%CI: 63.1–81.8%) (red line) for patients with negative mpMRI. Abbreviations: bNED: biochemical no evidence of disease; sRT: salvage radiotherapy; VUA: vesico-urethral anastomosis; BN: bladder neck; RV: retro-vesical space; DCE-MRI: dynamic-contrast-enhanced magnetic resonance imaging; yrs: years; mpMRI: multiparametric magnetic resonance imaging.

Figure 3 illustrates bNED-survival rates by both the location of the disease and the volume of the lesion at DCE-MRI.

Patients were stratified by the location into ‘none’, ‘VUA-only’, ‘BN/RV ± VUA’ and by the volume using the median value of 0.4 cc for patients with positive DCE-MRI. Patients with lesions > 0.4 cc or located at BN/RV were pooled. For patients with VUA-only disease up to 0.4 cc, the bNED rate at 4 yrs was 94.6% (95%CI: 80.2–98.6%) as opposed to 80.9% (95%CI: 71.6–87.4%) and 73.7% (95%CI: 63.1–81.8%) for patients with other detectable lesions and no macrodisease, respectively. 

## 4. Discussion

In a recent retrospective study from Mayo University of 386 patients with biochemical failure after radical prostatectomy who underwent restaging with DCE-MRI prior to sRT, biochemical failure was less likely in patients with presumed local disease at DCE-MRI compared to those with negative findings [9]. Similarly, Song et al. [11] found that the absence of a visible lesion on DCE-MRI was a risk factor for sRT failure in a cohort of 149 men with biochemical failure after RP. Of note, according to both studies, the risk reduction in biochemical failure for patients with any abnormal local finding at DCE-MRI was found to be ranging from 42% to 52% [9,11] that is remarkably similar to the one found in the present study at MVA (Table 2). Moreover, here, we show that both the location of the recurrent disease within the prostatic fossa and its (aggregate) volume are independent predictors of surviving without a biochemical failure after sRT.

DCE-MRI allows identification with a high sensitivity and specificity (both around 90% even at PSA values consistent with early salvage) of the precise anatomic subsite of recurrence, which is critical to defining the most effective salvage treatment [19]. In our experience, small VUA-only recurrence have an excellent prognosis with bNED rates at 4 years of 94.6% (95%CI: 80.2–98.6%).

The fact that patients with presumed local disease at DCE-MRI have a better prognosis after local treatment than patients without it supports the hypothesis that the lack of a visible though unproven local source of PSA production is associated with a higher risk of micrometastatic disease elsewhere, regionally and/or distantly, at the time of sRT. For such patients, sRT has a higher probability of being futile since it may not cover all the disease. This is not a novel concept [20]. However, compared to other previous studies using DCE-MRI [9,11], the present one is unique in the fact that all the patients were restaged also with PET/CT to rule out distant or regional disease before sRT, which is in agreement with current guidelines [21]. Indeed, about 5–20% of patients are found to have disease outside the prostatic fossa on PET/CT before sRT [22,23], and their prognosis is significantly worse than the one for patients with normal PET/CT findings [22,24,25]. Unfortunately, due to the recognized poor sensitivity of PET imaging at lower PSA values, our data indirectly support the hypothesis that a percentage of the patients harbor extraprostatic fossa disease despite a negative PET/CT. Therefore, the predictive value of DCE-MRI holds true also in the context of a negative PET/CT. 

Moreover, among patients with presumed local disease at DCE-MRI, the risk of failing sRT is higher for patients with lesions above the VUA. In a previous study, we found no difference in the response rates at serial DCE-MRI among different tumor locations within the prostatic fossa [16]. Therefore, recurrent tumor location per se does not seem to support any difference in response to sRT, while it more likely reflects the worse prognosis of disease spread more distantly than the VUA. In a recent retrospective study on PSMA PET/CT, Dundee and colleagues [26] described the recurrence at the prostatic vascular pedicle, which includes the seminal vesicle, to be associated with a higher probability of extrapelvic disease progression (pelvic lymphnodes recurrence or pelvic bones metastasis) compared to patients with VUA recurrence, supporting our results of better bNED rates for VUA-only lesions.

The finding that larger (aggregate) lesions have a worse prognosis can be interpreted in terms of both a lower response rate [16] and a higher probability of extraprostatic fossa disease at increasing volume. 

Another interesting observation is that, contrary to expectations [27] and similarly to Schmidt Hegemann et al. [22], we did not find any added value of PSMA PET/CT over Choline PET/CT, though the number of patients who underwent PSMA PET/CT is somewhat small.

Finally, in the present paper, similarly to Zilli et al. [8], DCE-MRI was used to boost the site(s) of presumed local disease. This allowed selective dose escalation to areas of suspected local disease and results at 4 years are encouraging.

Even though DCE-MRI is not routinely performed to restage PCa patients with biochemical recurrence here, we have shown the important prognostic value of this exam in this setting. In particular, the volume and subsite of the local lesion seem to be correlated with higher rates of biochemical survival (bNED-rate at 4 yrs was 94.6%). Our results support the use of DCE-MRI for restaging as a prognostic tool that may help to identify those patients who will benefit more from sRT. 

However, the present paper has some limitations. First, imaging tests were performed during routine clinical activity and data were collected retrospectively, though a significant number of patients (N = 62) were treated within a prospective study [12] and another 59 were included in another cross-sectional study [15]. Second, delivered treatment was not homogeneous, particularly with regard to pelvic node inclusion, prostatic fossa dose prescription, and androgen deprivation association. We have previously reported our criteria for pelvic node inclusion [15], while the dose to the prostatic fossa was kept below 69 Gy in a few selected patients without local disease (Table 1). Regarding androgen deprivation, due to the lack of guidelines at the time patients were treated, only a minority of them underwent AD, which is indeed a strength of the paper. 

Other strengths include the fact that the time of accrual in our study is about half of the one of the Mayo Clinic one [9]; all patients underwent restaging with PET/CT before sRT, and all DCE-MRI images were reviewed by the same radiologist. 

## 5. Conclusions

Local DCE-MRI is recommended in all patients at restaging for biochemical failure after RP for prostate cancer since it can provide both predictive and therapeutic information. Patients with small lesions located at the VUA have an excellent prognosis after sRT alone.

## Figures and Tables

**Table 1:** Selected patients, tumor and treatment characteristics.

Characteristic	Stratification	Median/#	IQR/%
Age	Continuum	69.4	66–75
pT stage at RP	2	135	57.2
	3a	84	35.6
	3b	17	7.2
pN stage at RP	pN0	136	57.6
	pNx	100	42.4
Surgical margins at RP	0	117	49.6
	1	115	48.7
	Missing	4	1.7
ISUP category at RP	1	20	8.5
	2	115	48.7
	3	71	30.1
	4	16	6.8
	5	14	5.9
Detectable PSA after RP	No	214	90.7%
	Yes	22	9.3%
Time from RP to sRT (mths)	Continuum	44.9	19.9–84.1
PSA at RP (ng/mL)	Continuum	7.2	5.3–10.0
Pre sRT PSA (ng/mL)	Continuum	0.51	0.35–0.96
PSADT (mths)	Continuum	10.8	4.5–20.1
Macro tumor load (cc) per pt	Continuum	0.41	0.22–0.83
# Lesions	0	90	38.1
	1	125	53.0
	2	20	8.5
	3	1	0.4
Involved subsites @ DCE-MRI	VUA	85	36%
	BN	39	16.5%
	RV	34	14.4%
DCE-MRI findings	Negative	90	38.1%
	Positive	146	61.9%
DCE-MRI	In-house	191	80.9%
	Outside Institution	45	19.1%
PET/CT	Choline only	144	61.0%
	Cu/PSMA ± Choline	92	39.0%
WPRT	No	86	36.4%
	Yes	150	63.6%
EAU risk	Low	88	37.3%
	High	144	61.0%
Androgen Deprivation	None	211	89.4%
	Any	25	10.6%
Dose to the prostatic fossa	66 Gy	40	16.9%
	69 Gy	196	83.1%

Abbreviations: #: number; IQR: interquartile range; pT: pathological tumor stage; RP: radical prostatectomy; pN: pathological nodal stege; ISUP: International Society of Urological Pathology; PSA: prostate-specific antigen; mths: months; sRT: salvage radiotherapy; PSADT: PSA doubling time; pt: patient; DCE-MRI: dynamic-contrast-enhanced magnetic resonance imaging; PET/CT: positron emission tomography/computed tomography; WPRT: whole pelvis radiotherapy; EAU: European Association of Urology.

**Table 2:** Multivariable Analysis.

		Positive vs. Negative DCE-MRI			Disease Location at DCE-MRI		
Covariate	Stratification	HR	95%CI	*p* Value	HR	95%CI	*p* Value
SVI at RP	No	1			1		
	Yes	2.84	1.18–6.84	0.020	2.44	0.99–5.96	0.051
DCE-MRI status	Negative	1					
	Positive	0.52	0.29–0.95	0.035			
Location at DCE-MRI	None				1		
	VUA only				0.34	0.15–0.77	0.010
	BN/RV ± VUA				0.73	0.37–1.42	0.319
Total Volume (cc)	Continuum	1.06	1.02–1.11	0.006	1.07	1.02–1.12	0.003
EAU risk	Low	1			1		
	High	2.03	0.99–4.16	0.052	2.06	1.01–4.21	0.048

Abbreviations: DCE-MRI: dynamic contrast-enhanced-magnetic resonance imaging; HR: hazard ratio; 95%CI: 95% confidence interval; SVI: seminal vesicle invasion; RP: radical prostatectomy; EAU: European Urology Association.

## Data Availability

Data are available at https://gbox.garr.it/garrbox/index.php/s/dqPpMoUtgefBAsF (accessed on 1 February 2023).

## Supplementary Materials

The following supporting information can be downloaded at: https://www.mdpi.com/article/10.3390/cancers15041246/s1, Table S1: Univariable analysis on bNED survival.
