# Supplementary material for: The Prognostic Value of DCE-MRI Findings before Salvage Radiotherapy after Radical Prostatectomy
Source: Cancers (Basel). 2023 Feb 15;15(4):1246. doi: 10.3390/cancers15041246 (PMC9954112; doi:10.3390/cancers15041246)
Supplement: Supplementary file 1 [file cancers-15-01246-s001.zip › cancers-2120184-supplementary.pdf]

Supplementary Table S1. Univariable analysis on bNED survival.

Covariate	Stratification	HR	95%CI	p value
Age	Continuum	1.01	0.97-1.05	0.612
pT stage	2	1		
	3	1.27	0.72-2.22	0.405
SVI	No	1		
	Yes	2.48	1.11-5.5	0.026
Surgical margins at RP	R0	1		
	R1	1.08	0.62-1.89	0.786
ISUP category	1-2	1		
	3-5	1.38	0.79-2.42	0.255
PSA detectable after RP	No	1		
	Yes	1.79	0.81-3.99	0.152
Time from RP to sRT (mths)	Continuum	1.00	0.99-1.00	0.426
PSA at RP (ng/ml)	Continuum	1.01	1.00-1.03	0.061
Pre sRT PSA (ng/ml)	Continuum	1.10	0.94-1.28	0.227
PSADT (mths)	Continuum	0.97	0.94-0.99	0.021
Multiparametric MR	Negative	1		
	Positive	0.605	0.345-1.062	0.080
# local lesions	None	1		
	1	0.62	0.35-1.11	0.111
	2+	0.49	0.15-1.63	0.245
Sites	None	1		
	VUA only	0.38	0.18-0.82	0.014
	BN/RV±VUA	0.88	0.47-1.65	0.694
Total Volume (cc)	Continuum	1.06	1.01-1.10	0.014
DCE-MRI	In	1		
	Out	0.74	0.35-1.58	0.439
PET/CT	Choline only	1		
	Cu/PSMA±Choline	0.82	0.45-1.47	0.500
WPRT	No	1		

	Yes	0.96	0.54-1.72	0.896
EAU risk	Low	1		
	High	2.38	1.21-4.67	0.012
Androgen deprivation	None	1		
	Any	1.87	0.91-3.86	0.090

Abbreviations: bNED: biochemical no evidence of disease; HR: hazard ratio; 95%CI: 95% confidence interval; pT: pathological tumor stage; SVI: seminal vesicle invasion; RP: radical prostatectomy; ISUP: International Society of Urological Pathology; PSA: prostate specific antigen; sRT: salvage radiotherapy; mths: months; PSADT: PSA doubling time; MR: magnetic resonance; VUA: vesico-urethral anastomosis; BN: bladder neck; RV: retro-vesical space; DCE-MRI: dynamic contrast-enhanced-magnetic resonance imaging; PET/CT: positron emission tomography/compted tomography; PSMA: prostate specific membrane antigen; WPRT: whole pelvis radiotherapy; EAU: European Urology Association;
